# After total hip arthroplasty, patients with glucocorticoid-treated osteonecrosis of the femoral head have a higher risk of in-hospital reoperation: a nationwide propensity score-matched analysis

**DOI:** 10.1007/s00264-026-06875-3

**Published:** 2026-06-04

**Authors:** Hidetatsu Tanaka, Kunio Tarasawa, Yu Mori, Kazuyoshi Baba, Hideki Fukuchi, Hiroki Kawamata, Kiyohide Fushimi, Toshimi Aizawa, Kenji Fujimori

**Affiliations:** 1 Department of Orthopaedic Surgery, Tohoku University Graduate School of Medicine, Sendai, Japan; 2 Department of Medical Information Technology Center, Tohoku University Hospital, Sendai, Japan; 3 Department of Health Policy and Informatics, Institute of Science Tokyo, Tokyo, Japan; 4 Tohoku University, Sendai, Japan

**Keywords:** Osteonecrosis of the femoral head, Glucocorticoid, Total hip arthroplasty, Post-operative complications, Large data base

## Abstract

**Purpose:**

Inpatient glucocorticoid treatment has been associated with increased postoperative complications after total hip arthroplasty (THA). However, evidence specific to osteonecrosis of the femoral head (ONFH) remains limited and inconsistent. This study aimed to evaluate the association between inpatient glucocorticoid treatment and early postoperative complications after THA for ONFH using a nationwide database.

**Methods:**

This retrospective cohort study used the Japanese Diagnosis Procedure Combination database from 2011 to 2023. Patients undergoing THA for ONFH were identified and categorized according to inpatient glucocorticoid treatment. Propensity score matching was performed using demographic and perioperative variables. The primary outcome was reoperation during hospitalization. Secondary outcomes included surgical and medical complications and in-hospital mortality. Multivariate logistic regression analysis was performed to identify independent risk factors.

**Results:**

After propensity score matching, 19,154 patients were included. Inpatient glucocorticoid treatment was independently associated with an increased risk of reoperation (odds ratio [OR], 1.73; 95% confidence interval [CI], 1.26–2.37; *p* < 0.001). No significant associations were observed between inpatient glucocorticoid treatment and dislocation (OR, 1.17; *p* = 0.45), surgical site infection (OR, 1.03; *p* = 0.86), periprosthetic fracture (OR, 1.38; *p* = 0.24), or in-hospital mortality (OR, 1.69; *p* = 0.35). Medical complications were also not significantly different between the groups. Closed reduction and revision arthroplasty were the most common reoperations in both groups.

**Conclusion:**

Patients receiving inpatient glucocorticoid treatment had a higher risk of reoperation after THA for ONFH, despite no significant increase in most early postoperative complications. These findings suggest that patients receiving inpatient glucocorticoid treatment may represent a medically vulnerable population at increased risk of early postoperative reoperation after THA. Careful perioperative management may be warranted in this high-risk population.

## Introduction

Osteonecrosis of the femoral head (ONFH) is an important and relatively common hip disease that affects younger and medically complex patients. In Japan, approximately 11,400 patients are treated annually, with approximately 2,200 new cases each year [1], and more than half require surgical intervention within  nine months of diagnosis, including joint replacement surgery (67.5%), given that spontaneous remission is uncommon [2]. The condition is characterized by progressive femoral head collapse, leading to secondary osteoarthritis, severe hip pain, and substantial limitations in activities of daily living [3–5]. The aetiology of ONFH is multifactorial and includes trauma, glucocorticoid use, alcohol abuse, autoimmune diseases, and other pathogenic mechanisms [6–9]. Glucocorticoid exposure is a well-recognized risk factor and is frequently observed in patients with systemic inflammatory and immune-mediated conditions, such as systemic lupus erythematosus, vasculitis, nephrotic syndrome, hematologic disorders, and inflammatory bowel disease [10–12]. In addition to its etiologic role, patients receiving glucocorticoid treatment often have systemic inflammatory diseases, immunosuppression, frailty, osteoporosis, or impaired tissue quality, which may contribute to increased postoperative vulnerability after THA [13–15]. Accordingly, glucocorticoid use has been suspected to increase the risk of early postoperative complications after total hip arthroplasty (THA). Compared with patients undergoing THA for primary osteoarthritis, those with ONFH are generally younger, have greater systemic comorbidity, and more frequently require immunosuppressive therapy, including glucocorticoids, before surgery [16–18]. These differences are clinically important, as THA for ONFH has been associated with a higher risk of revision than THA for osteoarthritis, although findings regarding dislocation and functional outcomes remain inconsistent across studies [16, 17, 19–21].

Previous studies have suggested that preoperative corticosteroid use is associated with an increased risk of infectious complications after total joint arthroplasty. In THA, corticosteroid use has been linked to higher risks of sepsis, surgical site infection, periprosthetic joint infection, and hospital readmission [13, 15, 22]. Corticosteroid use within one year before THA has also been associated with an increased risk of prosthetic joint infection, independent of underlying disease [12]. However, the current literature remains heterogeneous and incomplete, with some studies reporting no independent association between corticosteroid use and postoperative complications after total joint arthroplasty [23]. Many studies have evaluated mixed arthroplasty populations or selected disease cohorts, making it difficult to isolate the specific impact of glucocorticoid use in ONFH [12, 14, 21]. In addition, ONFH-specific studies are limited by small sample sizes and inconsistent findings regarding postoperative outcomes [21, 24].

Therefore, the purpose of this study was to evaluate the association between inpatient glucocorticoid treatment and early postoperative complications after total hip arthroplasty (THA) for osteonecrosis of the femoral head (ONFH) using a large nationwide database. We hypothesized that glucocorticoid use would be independently associated with an increased risk of reoperation after THA for ONFH.

## Patients and methods

### Study design and setting

This retrospective cohort study utilized data from the Japanese Diagnosis Procedure Combination (DPC) database and was conducted in accordance with the principles of the Declaration of Helsinki. Ethical approval was obtained from the Institute of Science Tokyo (Approval No. M2000-788) and the Tohoku University Graduate School of Medicine (Approval Nos. 2021–1–1082 and 2024–1–1026). As the DPC database contains anonymized data, the requirement for informed consent was waived. Data for this study were obtained from the DPC database, as previously described [18].

The dataset covered the period from December 2011 to March 2023 and included clinical information from approximately 1,100 hospitals participating in the DPC system across Japan. The DPC database is an administrative claims and discharge abstract database, and is one of the largest nationwide inpatient databases in Japan. It contains anonymized patient-level data, including demographic characteristics, diagnoses coded according to the International Classification of Diseases, 10th Revision (ICD-10), admission and discharge dates, surgical and nonsurgical procedures, comorbidities, and in-hospital complications. Diagnoses and complications were identified using ICD-10 codes recorded in the DPC database.

### Participants and data selection

Patients who underwent total hip arthroplasty (THA) for osteonecrosis of the femoral head were identified using ICD-10 codes for osteonecrosis (M87.x) based on three primary criteria: (1) the main diagnosis, (2) the principal reason for hospitalization, and (3) the condition requiring the greatest intensity of medical resource utilization. Patients who underwent THA for other indications, including osteoarthritis, rheumatoid arthritis, and trauma-related conditions, such as proximal femoral or acetabular fractures, were excluded. In addition, patients with missing or incomplete data for key variables were excluded from the analysis. Underlying diseases requiring glucocorticoid therapy were categorized according to ICD-10 codes as follows: collagen diseases (M32–M35), including systemic lupus erythematosus (M32.9), vasculitis (M30–M31), leukaemia (C91–C95), and nephrotic syndrome (N04).

Between December 2011 and March 2023, a total of 26,821 eligible patients were identified. The analysis was restricted to resource-intensive cases, as defined by the DPC system, which represents the principal hospitalization records and reflects the real-world clinical practice of THA in Japan. In accordance with the guidelines of the Association of Anesthetists’ and the UK Endocrine Society, this study defined glucocorticoid use as the continuous administration of glucocorticoids at a dose exceeding 5 mg/day (prednisolone equivalent) throughout the hospitalization period [25–27]. Because the DPC database does not contain information regarding outpatient prescriptions, cumulative glucocorticoid exposure, or exact treatment duration before hospitalization, inpatient glucocorticoid administration exceeding 5 mg/day (prednisolone equivalent) was used only as a surrogate marker of ongoing glucocorticoid treatment at the time of surgery rather than a direct measure of chronic exposure. This definition may have included patients receiving perioperative stress-dose steroids, transient inpatient glucocorticoid treatment, or steroid administration for acute medical conditions. Conversely, chronic outpatient glucocorticoid users whose medications were not continued during hospitalization may not have been identified.

### Propensity score matching

**Fig. 1:**
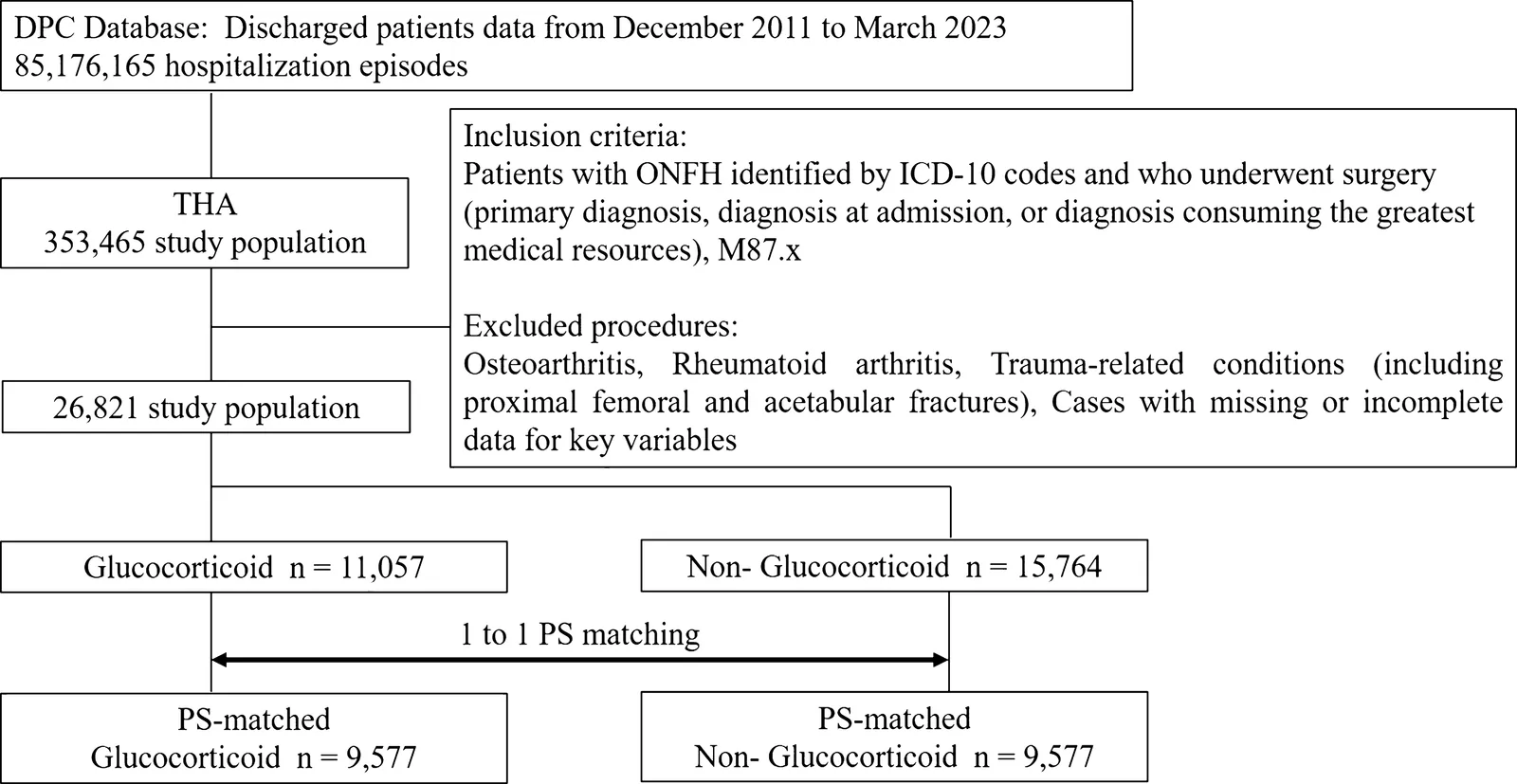
Study flow chart. DPC: Diagnosis procedure combination, THA: Total hip arthroplasty, ONFH: Osteonecrosis of the femoral head, ICD-10: International Classification of Diseases, 10th Revision, PS: Propensity score

This study compared postoperative complications between patients who underwent THA with and without glucocorticoid treatment. To minimize baseline differences between groups, propensity score (PS) matching was performed. The PS was estimated using a logistic regression model including the following covariates: age, sex, body mass index (BMI), unilateral or bilateral surgery, anesthesia type, Charlson Comorbidity Index (CCI), smoking, cement fixation, and use of computer-assisted orthopaedic surgery (CAOS). One-to-one nearest-neighbor matching without replacement was conducted with a caliper width of 0.2 times the standard deviation of the PS. Model discrimination was assessed using the C-statistic. After matching, 19,154 patients were included in each cohort. The C-statistic was 0.875, and all covariates demonstrated standardized mean differences (SMDs) of < 0.1, indicating adequate covariate balance (Table 1, Fig. 1).

**Table 1:** Baseline demographic data

		Before PS matching			After PS matching	
Covariate	GC group	Non-GC group	*P*-value	GC group	Non-GC group	SMD
n	11057	15764		9577	9577	
Age	59.1 ± 15.0	60.6 ± 14.6	< 0.001※	60.1 ± 14.6	60.1 ± 14.8	0.003
Gender (%)						
Men	4341 (39.3)	8394 (53.3)	< 0.001	4177 (43.6)	5400 (56.4)	0.017
Women	6716 (60.7)	7370 (46.7)		4098 (42.8)	5479 (57.2)	
BMI	23.2 ± 4.1	23.3 ± 4.0	0.167※	23.2 ± 4.0	23.2 ± 4.1	0.004
Surgical side (%)						
Unilateral	10736 (97.1)	321 (2.9)	< 0.001	9341 (97.5)	9354 (97.7)	0.009
Bilateral	15456 (98.1)	308 (1.9)		236 (2.5)	223 (2.3)	
General anesthesia	8092 (73.2)	12090 (76.7)	< 0.001	7120 (74.4)	7103 (74.2)	0.004
CCI	0.98 ± 1.12	0.64 ± 1.03	< 0.001※	0.84 ± 1.02	0.82 ± 1.15	0.017
Smoking	4074 (36.9)	6772 (43.0)	< 0.001	3735 (39.0)	3712 (38.8)	0.005
Use of bone cement	1785 (16.1)	2517 (16.0)	0.698	1553 (16.2)	1525 (15.9)	0.007
CAOS	3097 (28.0)	3704 (23.5)	< 0.001	2564 (26.8)	2601 (27.2)	0.009
Noncovariate	GC group	Non-GC group	*P*-value	GC group	Non-GC group	*P*-value
Length of hospital stay	25.6 ± 16.3	26.7 ± 15.8	< 0.001※	25.5 ± 16.4	26.6 ± 15.8	< 0.001
Medications (%)						
Anticoagulate agent	7891 (71.4)	11,282 (71.6)	0.721	6858 (71.6)	6812 (71.1)	0.472
Antiplatelet agent	1081 (9.8)	1134 (7.2)	< 0.001	886 (9.3)	747 (7.8)	< 0.001
Comorbidities (%)						
Hypertension	1729 (15.6)	2366 (15.0)	0.163	1540 (16.1)	1472 (15.4)	0.184
Diabetes	1630 (14.7)	1632 (10.4)	< 0.001	1286 (13.4)	1175 (12.3)	< 0.05
Nephrotic syndrome	255 (2.3)	57 (0.4)	< 0.001	221 (2.3)	37 (0.4)	< 0.001
leukemia	85 (0.8)	56 (0.4)	< 0.001	63 (0.7)	56 (0.6)	0.581
SLE	1101 (10.0)	127 (0.8)	< 0.001	743 (7.8)	118 (1.2)	< 0.001
Vasculitis	199 (1.8)	14 (0.1)	< 0.001	184 (1.9)	7 (0.1)	< 0.001
Other collagen diseases	930 (8.4)	111 (0.7)	< 0.001	748 (7.8)	88 (0.9)	< 0.001

Age, BMI, CCI, and length of hospital stay are shown as mean ± standard deviation

*P*-values of < 0.05 are considered significant by the Student-t test andχ2 test

*GC* Glucocorticoid, *SMD* Standardized Mean Difference, *BMI* Boby Mass Index, *CCI* Charlson Cormobidity Index, *CAS* Computer-assisted orthopedic surgery, *SLE* systemic lupus erythematosus

※ Student-t test

### Outcomes

Surgical complications included postoperative dislocation, surgical site infection, periprosthetic fracture, wound dehiscence, and reoperation. Medical complications included hospital-acquired pneumonia, deep vein thrombosis (DVT), pulmonary embolism (PE), cardiac and cerebrovascular events, acute renal failure, and in-hospital mortality.

### Statistical analysis

Categorical variables were compared using the chi-squared test, and continuous variables were compared using Student’s t-test. Univariate and multivariable logistic regression analyses were performed within the propensity score-matched cohort to evaluate the association between glucocorticoid use and each postoperative complication. In addition, multivariable logistic regression analysis was conducted to identify factors independently associated with reoperation within the matched cohort. Furthermore, multivariable logistic regression analysis was performed to identify independent predictors of major postoperative complications, adjusting for factors other than glucocorticoid exposure. Data are presented as mean ± standard deviation (SD) or odds ratios (ORs) with 95% confidence intervals (CIs). Statistical significance was defined as p < 0.05, and standardized mean differences (SMDs) < 0.1 were considered indicative of adequate balance. All statistical analyses were performed using JMP version 19.0 (SAS Institute Inc., Cary, NC, USA).

## Results

### Surgical complications

The absolute reoperation rate was 2.32% in the glucocorticoid group and 1.43% in the non-glucocorticoid group. Glucocorticoid use was associated with a significantly higher risk of reoperation (OR, 1.73; 95% CI, 1.26 to 2.37; p < 0.001). No significant associations were observed between glucocorticoid use and dislocation (OR, 1.17; 95% CI, 0.78 to 1.76; p = 0.45), surgical site infection (OR, 1.03; 95% CI, 0.77 to 1.36; p = 0.86), periprosthetic fracture (OR, 1.38; 95% CI, 0.80 to 2.37; p = 0.24), or wound dehiscence (OR, 0.70; 95% CI, 0.32 to 1.53; p = 0.36) (Table 2).

**Table 2:** Association between use of glucocorticoid and complications

Complications			Univariate analysis			Multivariable analysis		
	n	OR	95% CI	*P-value*	OR	95% CI	χ2 statics	*P-value*
Dislocation	194	1.430	1.043 to 1.906	< 0.05	1.167	0.776 to 1.756	0.553	0.450
Infection	200	1.129	0.854 to 1.493	0.434	1.025	0.771 to 1.363	0.030	0.862
Periprosthetic fracture	61	1.776	1.052 to 2.997	< 0.05	1.377	0.800 to 2.372	1.361	0.243
Wound dehiscence	26	0.733	0.337 to 1.597	0.557	0.697	0.319 to 1.525	0.826	0.363
Reoperation	359	1.635	1.319 to 2.027	< 0.001	1.730	1.264 to 2.366	12.11	< 0.001
Hospital-acquired pneumonia	58	1.524	0.890 to 2.580	0.147	1.475	0.868 to 2.505	2.106	0.147
DVT	836	0.932	0.812 to 1.071	0.340	0.929	0.809 to 1.068	1.074	0.300
PE	33	1.200	0.605 to 2.383	0.728	1.190	0.598 to 2.366	0.245	0.620
Cardiac event	5	0.667	0.111 to 3.990	0.999	0.565	0.092 to 3.475	0.389	0.533
Cerebrovascular event	41	1.564	0.835 to 2.931	0.211	1.520	0.809 to 2.286	1.730	0.188
Acute renal failure	6	0.500	0.092 to 2.730	0.688	0.510	0.093 to 2.788	0.639	0.424
Mortality during hospitalization	14	1.801	0.603 to 5.375	0.424	1.693	0.555 to 5.169	0.888	0.346

*P*-values of < 0.05 are considered significant by the χ2 test

*OR* Odds Ratio, *CI* Confidence Interval, *DVT* Deep Vein Thrombosis, *PE* Pulmonary Embolism

### Medical complications

No significant differences were observed between patients with and without glucocorticoid use in the incidence of hospital-acquired pneumonia (OR, 1.48; 95% CI, 0.87 to 2.51; p = 0.15), deep vein thrombosis (OR, 0.93; 95% CI, 0.81 to 1.07; p = 0.30), pulmonary embolism (OR, 1.19; 95% CI, 0.60 to 2.37; p = 0.62), cardiac events (OR, 0.57; 95% CI, 0.09 to 3.48; p = 0.53), cerebrovascular events (OR, 1.52; 95% CI, 0.81 to 2.29; p = 0.19), acute renal failure (OR, 0.51; 95% CI, 0.09 to 2.79; p = 0.42), or in-hospital mortality (OR, 1.69; 95% CI, 0.56 to 5.17; p = 0.35) (Table 2).

### Predictors of complications

Multivariate logistic regression analysis identified independent predictors of reoperation (Table 3). Glucocorticoid use was an independent predictor of reoperation (OR, 1.73; 95% CI, 1.26 to 2.37; p < 0.001). Older age (OR, 1.01; 95% CI, 1.00 to 1.02; p < 0.01) and a higher CCI (OR, 1.16; 95% CI, 1.05 to 1.27; p < 0.01) were also associated with an increased risk of complications. In contrast, other factors, including sex, body mass index, anaesthesia type, smoking status, hypertension, diabetes mellitus, nephrotic syndrome, leukaemia, systemic lupus erythematosus, vasculitis, and other collagen diseases, were not significantly associated with postoperative complications. To further characterize the increased risk of reoperation in the glucocorticoid group, the types of reoperations performed during hospitalization were analyzed (Table 4). Closed reduction and revision arthroplasty were the most common reoperation procedures in both groups. Closed reduction accounted for 26.7% of reoperations in the glucocorticoid group and 19.8% in the non-glucocorticoid group, whereas revision arthroplasty accounted for 24.2% and 32.1%, respectively (Table 4). However, the distribution of reoperation procedures did not significantly differ between the glucocorticoid and non-glucocorticoid groups.

**Table 3:** Multivariate logistic analysis of risk factors for re-operation

Variable	OR	95% CI	χ2 statics	*P-value*
Age	1.011	1.003 to 1.020	7.480	< 0.01
Gender (Female)	1.326	0.592 to 0.960	5.223	< 0.05
BMI	0.996	0.970 to 1.023	0.066	0.797
General anesthesia	0.940	0.742 to 1.190	0.263	0.608
CCI	1.158	1.053 to 1.267	8.910	< 0.01
Smoking	1.083	0.852 to 1.375	0.421	0.516
Use of bone cement	1.026	0.776 to 1.355	0.031	0.859
CAOS	0.723	0.558 to 0.937	6.388	< 0.05
Hypertension	0.823	0.609 to 1.111	1.682	0.195
Diabetes	0.795	0.567 to 1.108	1.928	0.165
Nephrotic syndrome	1.287	0.599 to 2.767	0.389	0.533
Leukemia	0.000	-	5.306	0.213
SLE	0.885	0.520 to 1.505	0.210	0.647
Vasculitis	0.420	0.104 to 1.705	1.975	0.160
Other collagen diseases	0.734	0.433 to 1.252	1.387	0.239
Glucocorticoid	1.705	1.367 to 2.126	22.88	< 0.001

*P*-values of < 0.05 are considered significant by the χ2 test;

*OR* Odds Ratio, *CI* Confidence Interval, *BMI* Boby Mass Index, *CCI* Charlson Comobidity Index, *CAOS* Computer-assisted orthopedic surgery, *SLE* Systemic lupus erythematosus

**Table 4:** Types of reoperation performed after THA

Type of reoperation	GC group n (%)	Non-GC group n (%)	OR	95% CI	*P-value*
Debridement/irrigation	15 (4.2)	8 (2.2)	1.168	0.482 to 2.833	0.827
Closed reduction	96 (26.7)	71 (19.8)	0.708	0.462 to 1.086	0.128
Open reduction	9 (2.5)	6 (1.7)	0.923	0.321 to 2.651	0.999
Revision arthroplasty	87 (24.2)	44 (32.1)	1.362	0.869 to 2.133	0.214
ORIF	15 (4.2)	8 (2.2)	1.168	0.482 to 2.833	0.827

*P*-values of < 0.05 are considered significant by the χ2 test

*OR* Odds Ratio, *CI* Confidence Interval, *ORIF* Open reduction and internal fixation

## Discussion

The most important finding of this nationwide propensity score-matched study was that inpatient glucocorticoid treatment was associated with a higher risk of reoperation after THA for ONFH. In contrast, glucocorticoid use was not significantly associated with most individual early surgical or medical complications, or in-hospital mortality. In addition, analysis of the types of reoperation demonstrated that closed reduction and revision arthroplasty were the most common reoperations in both groups, although the distribution of reoperation procedures did not significantly differ between groups.

Our findings should be interpreted in the context of previous studies on corticosteroid use and arthroplasty outcomes. Several reports have demonstrated that corticosteroid use is associated with an increased risk of infectious complications after joint arthroplasty, including periprosthetic joint infection, sepsis, and readmission [12–15]. In addition, glucocorticoid use has been linked to increased risks of pneumonia and in-hospital mortality in patients who undergo total knee arthroplasty in the DPC database [27]. In contrast, we did not observe significant associations between glucocorticoid use and individual coded postoperative complications such as dislocation, surgical site infection, or periprosthetic fracture. This discrepancy may partly reflect the limitations of diagnosis-based complication coding in administrative databases, whereas reoperation represents an actual unplanned procedural intervention and is widely regarded as a clinically meaningful arthroplasty endpoint [28, 29]. Therefore, our findings suggest that glucocorticoid-treated patients may be more likely to experience clinically important postoperative failure requiring additional intervention.

Patients receiving inpatient glucocorticoid treatment may represent a population with greater systemic disease burden, immune dysregulation, frailty, nutritional impairment, osteoporosis, or renal dysfunction, all of which may contribute to increased postoperative vulnerability [13–15, 22]. ONFH is not only a local disorder but is also associated with systemic abnormalities in bone marrow and vascular function, which may impair bone repair and regeneration [30]. These systemic effects may not be fully captured as individual coded complications during the index admission but may nevertheless increase the need for unplanned additional procedures. In our analysis, closed reduction and revision arthroplasty were the most common reoperations, both of which are frequently related to postoperative instability. Thus, the increased reoperation rate observed in glucocorticoid users may partly reflect instability-related events, although the precise clinical indications for reoperation could not be determined from the database. However, because the DPC database captures procedures rather than specific indications, the exact causes of reoperation could not be determined. Accordingly, the increased risk of reoperation observed in glucocorticoid-treated patients may partly reflect the severity of underlying systemic disease, frailty, or immunosuppressed status rather than the direct biological effects of glucocorticoids alone.

This interpretation is partly consistent with prior ONFH- and immunosuppression-related THA literature. THA performed for ONFH has been associated with a higher risk of revision than THA for osteoarthritis [20, 31], likely reflecting differences in patient age, activity level, bone quality, and systemic disease burden [32]. Rahman et al. reported a 17% complication rate and an 11% reoperation rate after THA in corticosteroid-induced osteonecrosis, suggesting increased postoperative vulnerability in this population [33]. Similarly, patients with systemic inflammatory diseases have been reported to have higher risks of postoperative complications after THA, including dislocation, infection, and readmission [21, 34]. Overall, these findings suggest that glucocorticoid-treated patients may represent a medically vulnerable population with increased postoperative risk, although the observed associations are likely influenced by underlying systemic disease burden and immunosuppression.

Although glucocorticoid use was not significantly associated with coded dislocation events, instability may still have contributed to the increased reoperation rate because closed reduction and revision arthroplasty were among the most common reoperations. Previous studies have shown that patients undergoing THA for ONFH have a higher risk of instability-related complications than those with osteoarthritis [18, 35–37]. In addition, CAOS was associated with a lower risk of reoperation in our study, consistent with prior reports demonstrating reduced instability-related complications with improved component positioning [38–41]. Similarly, prior studies in rheumatoid arthritis and other immunosuppressed arthroplasty populations have demonstrated increased risks of postoperative infection, instability, revision, and readmission after THA, supporting the concept that systemic inflammatory burden and immunosuppression may contribute to postoperative vulnerability independent of ONFH itself [21, 34].

We did not observe a significant association between glucocorticoid use and in-hospital mortality; however, this finding should be interpreted with caution. Previous studies have reported no significant difference in early postoperative mortality, including in-hospital or 30-day mortality [11, 14], whereas longer-term mortality has been shown to increase among glucocorticoid users [11]. Because our analysis was limited to in-hospital outcomes, longer-term mortality related to patients receiving inpatient glucocorticoid treatment and underlying systemic disease may not have been captured, and its impact may become more apparent over extended follow-up. Therefore, the increased reoperation risk observed in glucocorticoid-treated patients may partly reflect underlying systemic illness severity or immunosuppressed status rather than glucocorticoid exposure alone.

Large-scale epidemiological studies using the DPC database play an important role in orthopaedic research and the development of evidence-based clinical strategies [42–45]. However, several limitations inherent to the DPC database should be acknowledged. First, diagnoses and complications were identified using ICD-10 codes and recorded procedures, which may be subject to misclassification or coding errors. Second, detailed clinical information, such as imaging findings, extent of necrosis, and disease stage, was not available, limiting risk stratification. Important variables, including cumulative glucocorticoid exposure, outpatient medication history, laboratory data, surgical approach, implant characteristics, and surgeon experience, were also unavailable. Third, glucocorticoid exposure was defined based on inpatient administration exceeding 5 mg/day (prednisolone equivalent), which was used only as a surrogate marker of ongoing glucocorticoid treatment at the time of surgery. Because outpatient prescription data, cumulative exposure, and exact treatment duration were unavailable, exposure misclassification may have occurred. Patients receiving transient perioperative steroid supplementation may have been included, whereas chronic outpatient glucocorticoid users whose medications were not continued during hospitalization may not have been identified. The database could not distinguish whether glucocorticoid administration was preoperative, postoperative, or perioperative. Fourth, detailed timing and precise clinical indications for reoperation could not be fully evaluated because the administrative database contains procedural codes rather than detailed operative findings or clinical diagnoses. Furthermore, postoperative follow-up was limited to the index hospitalization period, and delayed complications or reoperations occurring after discharge could not be assessed. Finally, residual confounding may persist despite propensity score matching and multivariate adjustment. Therefore, the observed associations should be interpreted as non-causal. Future studies integrating administrative data with imaging, clinical staging, and patient-reported outcomes are needed to provide more comprehensive insights into ONFH management.

## Conclusion

In this nationwide propensity score-matched study, inpatient glucocorticoid treatment was associated with an increased risk of in-hospital reoperation after THA for ONFH. These findings suggest that glucocorticoid-treated patients may represent a medically vulnerable population. Careful perioperative management and surgical optimization may be warranted in this high-risk population, and further studies with detailed clinical data and longer follow-up are needed. However, these findings should be interpreted as associative rather than causal because of residual confounding and limitations in exposure definition inherent to administrative database studies.

## Data Availability

No datasets were generated or analysed during the current study.

## Abbreviations

*THA*: Total hip arthroplasty
*ONFH*: Osteonecrosis of the femoral head
*OR*: Odds ratio
*CI*: Confidence interval
*DPC*: Diagnosis procedure combination
*ICD-10*: International Classification of Diseases, 10th Revision
*PS*: Propensity score
*BMI*: Body mass index
*CCI*: Charlson comorbidity index
*CAOS*: Computer-assisted orthopedic surgery
*SMDs*: Standardized mean differences
*DVT*: Deep vein thrombosis
*PE*: Pulmonary embolization
*SD*: Standard deviation
